# Modification of low temperature magnetic interactions in Dy_1−*x*_Eu_*x*_MnO_3_

**DOI:** 10.1039/c8ra00224j

**Published:** 2018-04-11

**Authors:** K. Yadagiri, R. Nithya, Shilpam Sharma, A. T. Satya

**Affiliations:** Materials Science Group, Indira Gandhi Centre for Atomic Research, HBNI Kalpakkam – 603102 India nithya@igcar.gov.in

## Abstract

Solid solutions of rare earth ion (Eu^3+^) substituted DyMnO_3_, Dy_1−*x*_Eu_*x*_MnO_3_ (*x* = 0.0–1.0) have been synthesized by ceramic method. Powder X-ray diffraction revealed single phase nature of the compounds with orthorhombic structure. Contributions from the atomic vibrations to the observation of Raman bands have been established and assigned to symmetry stretching and anti symmetry stretching, bending and tilting modes. Raman band frequencies of tilting, asymmetric stretching and bending modes were found to decrease with increasing europium concentration showing softening. Transport studies revealed that all the compounds show semiconducting nature. While the end compounds display hopping process for electrical conduction, all the substituted compounds showed activated type of conduction, and activated energy was found to reduce with increase in *x*. Molar susceptibility of the substituted compounds for *x* = 0.1, 0.3 and 0.5 revealed an antiferromagnetic transition corresponding to Mn ions. The fitted Curie–Weiss temperatures also suggested the existence of antiferromagnetic interactions in all the materials. The magnetic field dependent magnetization at various temperatures revealed paramagnetic nature down to 8 K below which hysteresis loops are observed. The presence of strong ferromagnetic correlations between Dy and Mn spins through apical oxygen ions results in the large coercive fields. For temperatures above the antiferromagnetic temperature of manganese ions (39 K) *M*–*H* curves show almost straight lines implying the absence of ferromagnetic interactions in the compounds. Different magnetic transitions: from high temperature paramagnetic state to intermediate temperature antiferromagnetic state to low temperature ferromagnetic states are observed in the *M*–*H* data.

## Abstract 2

Solid solutions of rare earth ion (Eu^3+^) substituted DyMnO_3_, Dy_1−*x*_Eu_*x*_MnO_3_ (*x* = 0.0–1.0) have been synthesized by ceramic method.

## Graphical abstract

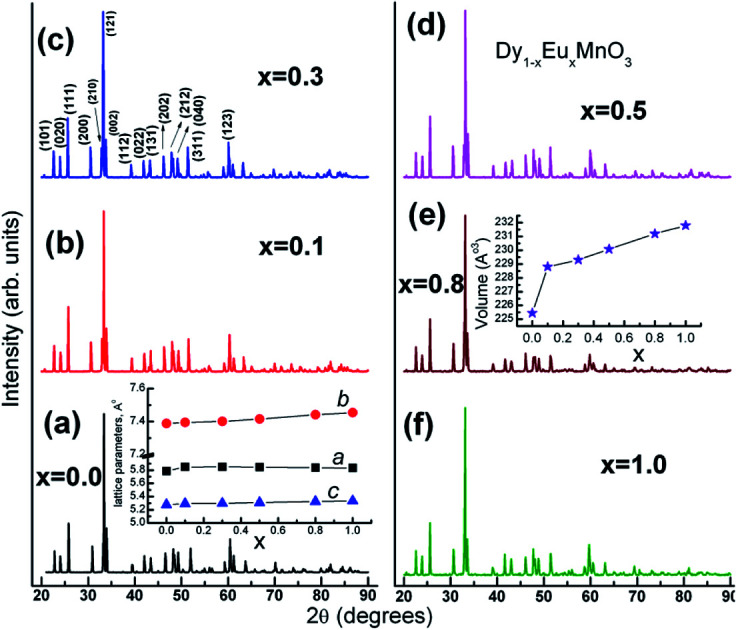

## Introduction

Perovskite oxides, ABO_3_ (A: alkali/rare earth ion and B: transition metal ion), are an important group of functional materials due to their diverse physical properties over a wide range of temperatures. Superconductivity, colossal magneto resistance, piezo/pyro/ferroelectricity and structural, magnetic and transport properties are some of the intriguing properties exhibited by these materials^1,2^ that attracted researchers attention from theoretical, experimental and application point of view. Among ABO_3_ family of oxides, rare earth (R) manganites, RMnO_3_ display spin dependent transport and magnetic properties^3–5^ thus making them good candidates for memory devices and spintronics applications. However, magnetic and transport properties are largely influenced by the ionic size of the R ion (*r*_R_). Kimura *et al.*^6^ reported a magnetic phase diagram as a function of *r*_R_. RMnO_3_ compounds adopt GdFeO_3_ perovskite structure where R and Mn atoms are deviated from the ideal positions in the cubic unit cell resulting in distortions of MnO_6_ octahedra with Mn–O–Mn bond angle less than 180 degrees. As a consequence of these changes, a series of magnetic transitions from high temperature paramagnetic to low temperature antiferromagnetic state with several spin arrangements^7,8^ have been observed. For example, in the case of orthorhombic DyMnO_3_ (DMO), high temperature paramagnetic phase changed to incommensurate sinusoidal antiferromagnetic (AFM) order (∼40 K) which in turn changed to commensurate magnetic structure (18 K) with the lowering of temperature.^7,9^ Whereas another member of the series, HoMnO_3_ with a smaller ionic radius at R site, displayed a commensurate magnetic structure that can be identified with “up-up-down-down” spin structure or E-type anti ferromagnetic structure.^10^ The modification of magnetic structures is understood to be due to the decrease of bond angle Mn–O–Mn in the crystal structure as a consequence of GdFeO_3_ – type distortions in addition to Jahn–Teller (JT) distortions. Yet in another member, YMnO_3_ with orthorhombic structure, frustrated antiferromagnetic ground state is observed.^11^ In particular, TbMnO_3_ and DyMnO_3_ have shown ferroelectric properties at low temperatures induced by spiral antiferromagnetic spin ordering of Mn ions from a high temperature sinusoidal antiferromagnetic incommensurate magnetic structure.^7^ Ferroelectricity is understood to be due to unconventional magnetic transition exhibited by DyMnO_3_ and TbMnO_3_ compounds. On other hand, EuMnO_3_ (EMO) with the same orthorhombic structure is reported to have antiferromagnetic ground state but without any magnetic induced ferroelectricity.^12^ Thus RMnO_3_ compounds show complex magnetic ground states and the magnetic interactions have been understood to be due to two types of interactions: super exchange interactions between nearest neighbor (NN) and next nearest neighbor (NNN) Mn ions.^13–15^ In view of the different properties shown by RMnO_3_ and related systems, we have undertaken Dy_1−*x*_Eu_*x*_MnO_3_ system to study the effects of larger isovalent ion, Eu (*r*_Eu_^3+^ (8) = 1.066 Å)^16^ substitution at Dy (*r*_Dy_^3+^ (8) = 1.027 Å) site on their physical properties. While DMO belongs to prototype magnetoelectric multiferroic materials, EMO does not show magnetic induced ferroelectric order. Both compounds have an orthorhombic distorted perovskite structure yet display different physical properties. Due to the diverse properties exhibited by both the end member manganites, it is interesting to study the ground state properties of the mixed compounds of Dy_1−*x*_Eu_*x*_MnO_3_ to understand the coupling between the magnetic and electric properties. Various experimental techniques were employed to characterize the solid solution and to measure their physical properties. In this paper, structural, transport, magnetic and spectroscopic properties of Dy_1−*x*_Eu_*x*_MnO_3_ (*x* = 0.0, 0.1, 0.3, 0.5, 0.8 and 1.0) are presented.

## Experimental details

Bulk materials of the solid solution series of Dy_1−*x*_Eu_*x*_MnO_3_ (*x* = 0.0, 0.1, 0.3, 0.5, 0.8 and 1.0) were synthesized by the conventional ceramic method from the raw oxides of Dy_2_O_3_, Eu_2_O_3_ and MnO_2_ as per the stoichiometry of the nominal compositions. The mixture was ground thoroughly and heated around 1000 °C for 12 hours with intermediate grinding. Subsequently, mixture was once again heated at 1300 °C for 12 hours. The mixture was then compressed into circular pieces which were sintered at 1300 °C for 24 hours to obtain dense pellets for resistivity measurements. X-ray diffraction (XRD) technique using STOE diffractometer (Germany) with Cu k_α_ radiation (1.5406 Å) was used to characterize the compounds as well as to determine their crystal structure. To determine the stoichiometry in the compounds, chemical analysis was carried out by Inductively Coupled Plasma-Optical Emission Spectroscopy (ICP-OES) technique. In order to avoid spectral interference from the rare earth ions in ICP-OES technique, Atomic Absorption Spectroscopy was used to analyze Mn. X-ray photoelectron spectroscopy technique was employed to determine the oxidation states of ions using SPECS Surface Nano Analysis GmbH (Germany) spectrometer equipped with a monochromatic Al k_α_ X-ray source (*hν* = 1486.7 eV) operating at 15 kV and 9.0 mA. Electron detection was done with a 9 channel train detector. A pressure of 1.2 × 10^−9^ mbar was maintained in the vacuum chamber during the measurement and the takeoff angle of the photoelectron was 57.4°. Before recording the XPS spectra, to remove adsorbed surface impurities, sample surfaces were cleaned by Ar ion sputtering with 1 keV energy. Raman spectra of the materials were recorded at room temperature with 514.5 nm Ar ion laser using InVia micro-Raman spectrometer (Renishaw, UK) in backscattering configuration. The effects of spin disorders induced by europium substitution on the transport properties are studied by measuring electrical resistivity as a function of temperature using four-probe method from room temperature (300 K) down to a low temperature depending on the resistance of the compounds. Magnetic properties were investigated from the magnetization measurements. The magnetization was measured both as a function of temperature and field using MPMS 3 Ever cool SQUID-VSM (Quantum Design) magnetometer. The temperature dependent dc magnetization data (*M*–*T*) were collected in both Zero Field Cooled (ZFC) and Field Cooled (FC) modes at selected magnetic fields in the range of 50 Oe to 10 kOe in the temperature range of 2–400 K. Magnetization with respect to magnetic field (*M*–*H*) was also recorded at different temperatures in the range between ±7 kOe.

## Results and discussion

**Fig. 1:**
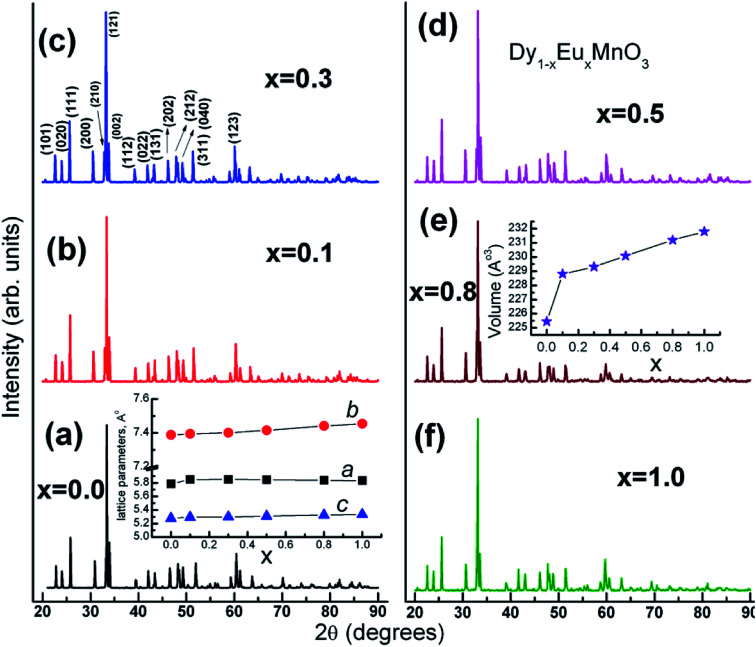
XRD patterns for Dy_1−*x*_Eu_*x*_MnO_3_ series of compounds. Dependence of unit cell parameters and volume with respect to europium concentration (*x*) are shown in separate insets.

Phase formation of the solid solution has been examined by X-ray powder diffraction technique. Fig. 1 shows XRD patterns of Dy_1−*x*_Eu_*x*_MnO_3_ (DEMO) (*x* = 0.0, 01, 0.3, 0.5, 0.8 and 1.0) compounds taken at room temperature. The diffraction peaks could be indexed to an orthorhombic structure using *Pnma* space group. In the solid solution series, the *Pnma* orthorhombic symmetry is retained. Lattice parameters were determined by Index and Refine program available with WinXPOW software provided by STOE (Germany) diffractometer. Miller indices, (*hkl*) corresponding to prominent diffraction peaks are marked for one of the compounds (*x* = 0.3) in the Fig. 1(c). Variation of the lattice parameters and cell volume as a function of europium concentration, *x* is shown in insets of the Fig. 1(a) and (e) respectively.

Results obtained from chemical analysis for the two substituted compounds, *x* = 0.1 and 0.3 along with the parent compound are tabulated in Table 1. Dy_0.5_Eu_0.5_MnO_3_ could not be dissolved by acid digestion method. As seen from the table, there is a good agreement between the chemical analysis results and calculated results by weight percentage as per the nominal composition.

**Table 1:** Elemental composition results obtained from chemical analysis given under exp. columns. Calculated values are also included in the table

Elements	Sample					
	DyMnO_3_ (wt%)		Dy_0.9_Eu_0.1_MnO_3_ (wt%)		Dy_0.7_Eu_0.3_MnO_3_ (wt%)	
	Exp.	Cal.	Exp.	Cal.	Exp.	Cal.
Dy	62.5	61	55.2	55	45.7	43.4
Eu	<0.3	0	6.0	5.8	19.7	17.4
Mn	20.8	21	19.5	20.8	18.9	21.0

**Fig. 2:**
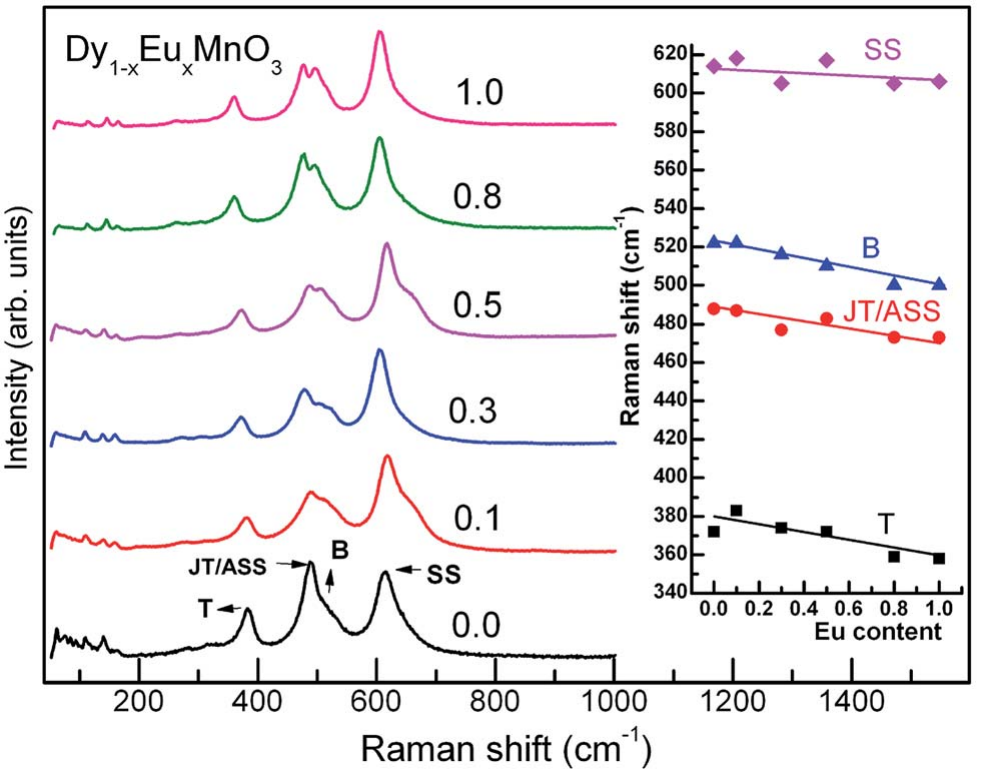
Raman spectra of Dy_1−*x*_Eu_*x*_MnO_3_. Inset shows the prominent mode frequencies variation with europium substitution at Dy site.

Raman spectra for all the compounds are illustrated in Fig. 2 (main panel). It is seen from the Fig. 2 that broad Raman bands observed between 200 and 1000 cm^−1^ are all internal modes and those below 200 cm^−1^ are associated with the lattice modes corresponding to heavy rare earth ions. Room temperature as well as temperature variation of Raman scattering spectra of manganites were reported by several authors^17–21^ where individual phonon modes have been correlated with structural distortions including Jahn–Teller ion (Mn^3+^) distortions. Raman spectra were fitted using Peakfit to obtain Raman mode frequencies. The mode frequencies agree with the reported values for similar manganites.^22^ Raman band frequencies of the JT/asymmetric stretching (ASS), tilting (T) and bending (B) modes decreased with increase in Eu concentration whereas that of symmetric stretching (SS) modes showed only a marginal decrease with increase in *x*. Inset of Fig. 2 shows the experimental findings of variation of Raman mode frequencies with europium concentration.

**Fig. 3:**
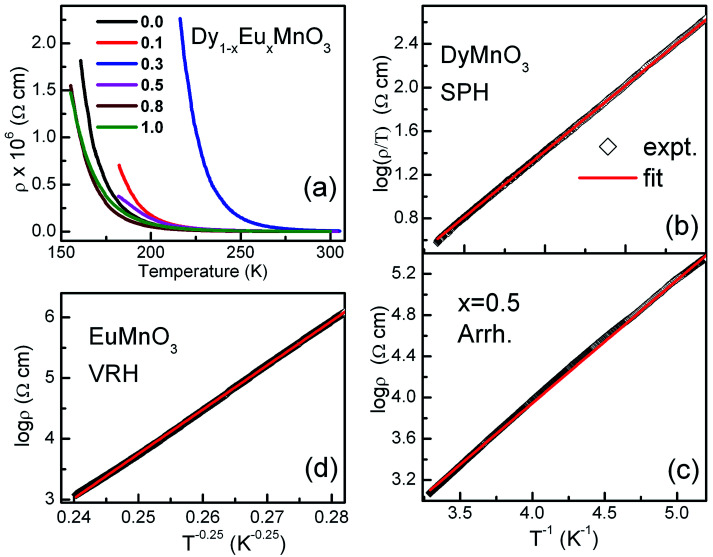
(a) Electrical resistivity of Dy_1−*x*_Eu_*x*_MnO_3_*versus* temperature for all the compositions of europium (b) SPH fitting for *x* = 0.0 (c) Arrhenius fitting for *x* = 0.5 (d) VRH fitting for *x* = 1.0.

Fig. 3(a) shows temperature dependence of electrical resistivity, *ρ*(*T*) of Dy_1−*x*_Eu_*x*_MnO_3_ from 150 K to 300 K. It is observed that the semiconducting behavior persists in the whole temperature range of measurement. In order to understand the charge transport mechanism for the observed semiconducting behavior, various models such as thermal activation process involving nearest neighbor hopping^23,24^ (Arrhenius law), hopping of small polarons (SPH) in the adiabatic approximation^25–27^ and variable range hopping^28^ (VRH) conduction process were used to fit the *ρ*(*T*) data. The equation for thermal activation process for nearest neighbor hopping conduction with activation energy (*E*_a_) is given by Arrhenius equation:1
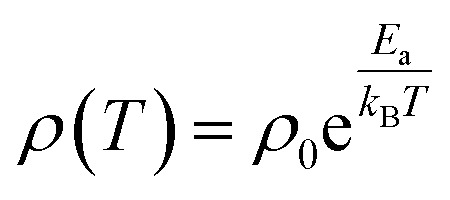


The equations for SPH and VRH conductions are:2
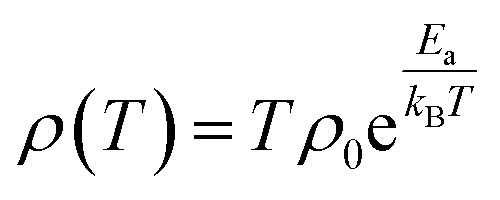
3
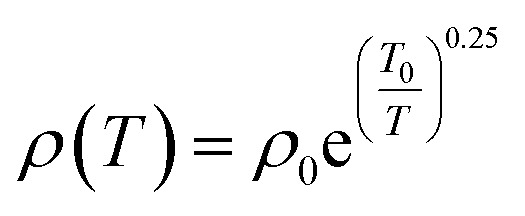


Fitting of the *ρ*(*T*) data to all the three models was carried out and the best fits showed that the nature of the electronic transport varied between the two end compounds and from the intermediate compounds as well. It was found that the conduction mechanism for the substituted compounds (*x* = 0.1 to 0.8) is dominated by the activated type of conduction in the whole temperature range whereas the end compounds DyMnO_3_ and EuMnO_3_ followed SPH and Mott type VRH mechanisms respectively. Linear fits of ln(*ρ*/*T*) with 1/*T*, ln *ρ* with 1/*T* and ln *ρ* with 1/*T*^1/4^ are shown in Fig. 3(b)–(d) respectively. The activation energies for the nearest neighbor hopping calculated from the fit parameters are: 110.0, 168.8, 104.4 and 94.4 meV for *x* = 0.1, 0.3, 0.5 and 0.8 respectively. Effect of europium substitution at Dy site on the activation energies for charge carriers can be presumably explained by changes in bond distances between Mn ions. Hopping amplitude of carriers between two Mn ions also decreases when the angle between the two Mn ions becomes smaller than 180 degrees. Thus the decrease in the activation energies can be interpreted in terms of the changes in the hopping distances. Moreover, carriers cannot find continuous paths between ions due to bending and tilting of MnO_6_ octahedra. In the composition regime 0.1 to 0.8, the hopping paths may be disturbed due to the probable disruption of the three dimensional network. This may be one of the reasons responsible for decreasing the conductivity in this composition range.

It may be noted that different transport mechanisms operate in the solid solutions Dy_1−*x*_Eu_*x*_MnO_3_. SPH was found in the pristine compound, DMO and then there is a crossover from SPH to activated behavior for the substituted compounds again to VRH for EuMnO_3_. Various parameters such as average ionic size, grain morphology, charge and structural disorders can directly affect the transport properties. Furthermore, increase in the resistivity with decrease in the temperature observed for all the compounds in the entire temperature range indicates a robust insulating behavior.

**Fig. 4:**
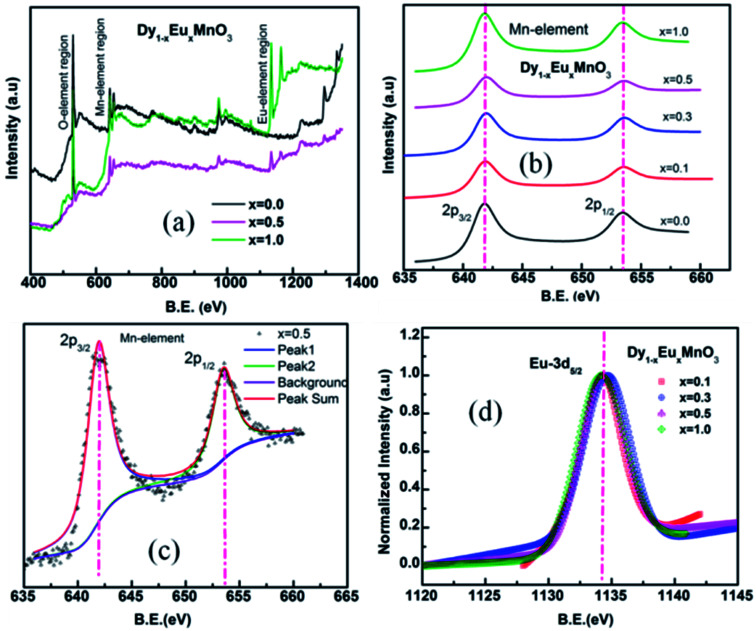
(a) Representative survey scan XPS spectra for the two end members and 50 at% substituted in the solid solution series. (b) Mn 2p spectra for all the compounds (c) deconvoluted spectra for *x* = 0.5 (d) normalized spectra for Eu 3d.

X-ray photoelectron spectra of Dy_1−*x*_Eu_*x*_MnO_3_ (*x* = 0.1–1.0) were recorded in the energy range 0–1000 eV. Representative survey scans corresponding to *x* = 0.0, 0.5 and 1.0 are depicted in Fig. 4(a). It is seen from the wide spectrum that all the elements are present in their respective binding energy scale. XPS spectra corresponding to manganese are shown in Fig. 4(b). Fig. 4(c) and (d) display a typical deconvoluted spectrum of Mn element for *x* = 0.5 and normalized spectra for DEMO for Eu 3d respectively. The binding energies determined from the fitting the spectra are tabulated in Table 2. Binding energies of Eu and Mn calculated from the fitting are found to be in agreement with the respective ions in the trivalent states.^29,30^ Binding energy of oxygen lies between 529.4–529.8 eV corresponding to O 1s.

**Table 2:** Binding energies of Dy_1−*x*_Eu_*x*_MnO_3_

*x*	Binding Energy (eV)				
	Eu		Mn		O
	3d_5/2_	3d_3/2_	2p_3/2_	2p_1/2_	1s
0.0	—	—	641.7	653.3	529.4
0.1	1134.33	—	641.8	653.5	529.6
0.3	1134.47	1163.99	641.8	653.5	529.8
0.5	1134.23	1163.68	641.9	653.5	529.7
1.0	1134.31	1164.05	641.7	653.4	529.6

XPS results indeed provide evidence for the existence of trivalent manganese and europium ions in all the substituted compounds in DEMO.

**Fig. 5:**
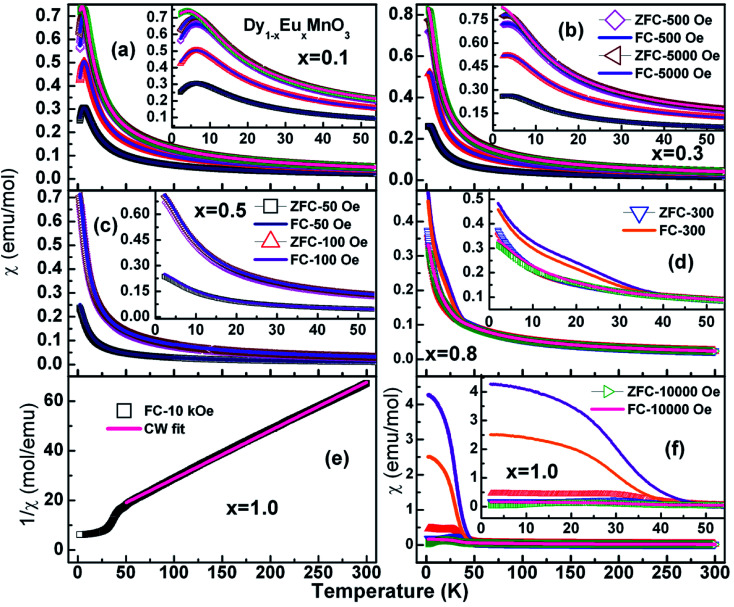
Molar susceptibility curves measured in the zero field cooled and field cooled cycles for DEMO compounds with *x* = 0.1 to 1.0 as mentioned in the respective graphs. The legends given are applicable to all the curves presented in various panels.

Earlier, Yadagiri *et al.*,^26^ reported the observation of three kinds of antiferromagnetic transition in the magnetization data of the pristine compound, DyMnO_3_. Following Kimura *et al.*,^7^ these transitions were attributed to an sinusoidal incommensurate antiferromagnetic ordering of Mn^3+^ ions (*T*_N1_ – 39 K), lock-in transition of Mn^3+^ ions (*T*_N2_ – 12 K) and antiferromagnetic ordering of Dy^3+^ ions (*T*_N_ – 3.62 K) respectively. Molar susceptibility (*χ*) of Dy_1−*x*_Eu_*x*_MnO_3_ (*x* = 0.1–1.0) as a function of temperature at different applied fields is displayed in Fig. 5.

**Fig. 6:**
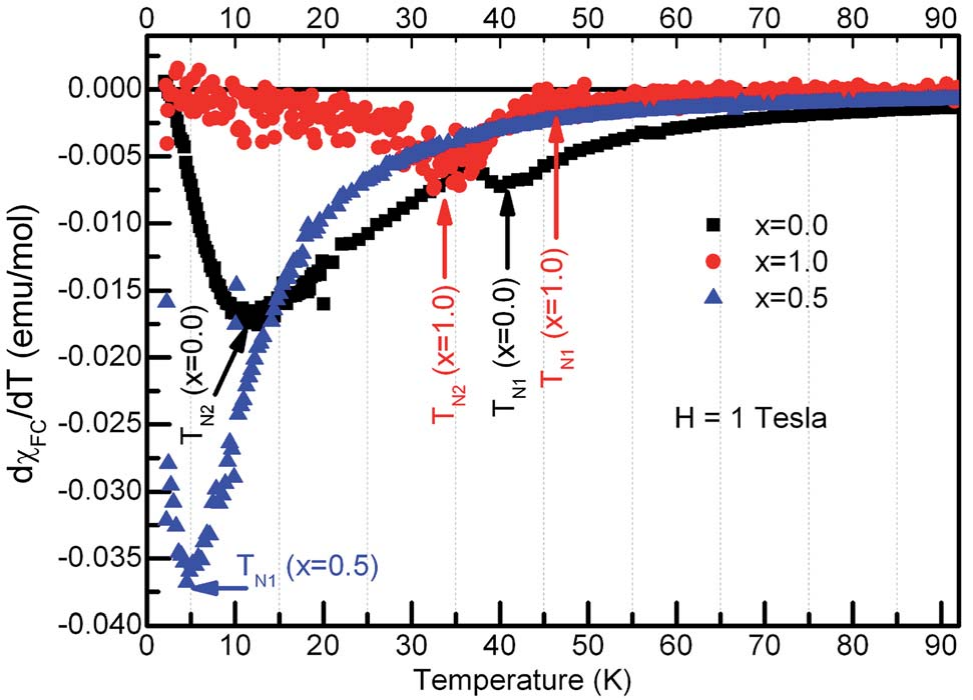
First derivative of molar susceptibility with temperature for the compounds, Dy_1−*x*_Eu_*x*_MnO_3_, *x* = 0.0, 0.5 and 1.0 as indicated in the figure.

It is seen from the Fig. 5 that the *χ*_ZFC_ and *χ*_FC_ curves measured under various strengths of the magnetic field overlap whose magnitude increased with decreasing the temperature in the entire temperature range for compounds with *x* = 0.1, 0.3 and 0.5 respectively. However, there appear broad peaks in both the *χ*_ZFC_ and *χ*_FC_ curves at all the fields whose peak temperature decreases slightly with field strength indicating the existence of a magnetic transition. Moreover, the temperature at which *χ*_ZFC_ and *χ*_FC_ curves bifurcate decreased when the external magnetic field increased. Cwik^31^ investigated magnetic and magnetoelectric properties of bulk compounds of (Dy_0.9_Er_0.1_)_1−*x*_Gd_*x*_Co_2_ (0.0 ≤ *x* ≤ 0.25). The irreversibility of the susceptibility between the ZFC and FC processes observed in our compounds is similar to that reported by Cwik. Further, the magnetic transition temperature is reduced with increasing the europium concentration, *x* up to *x* = 0.5. In view of the broader peaks, transition temperatures were identified from the minimum observed in the first derivative of the susceptibility curves, d*χ*_FC_/d*T versus* temperature. Thus the estimated transition temperatures are ∼12 K, ∼10 K and ∼5 K for *x* = 0.1, 0.3 and 0.5 respectively. Fig. 6 reports typical d*χ*_FC_/d*T* – *T* plots for selected compounds.

**Fig. 7:**
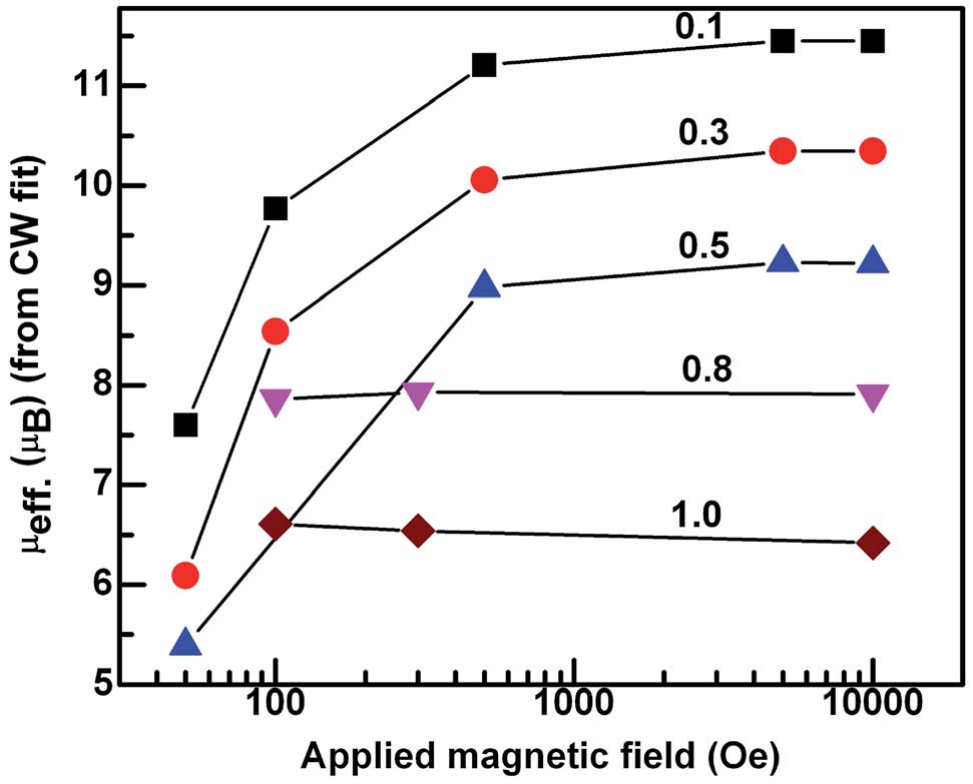
Semi log graph between the experimental effective magnetic moment *vs.* applied field for *x* in Dy_1−*x*_Eu_*x*_MnO_3_.

In the case of *x* = 0.8, (Fig. 5(d)) the *χ*_ZFC_ and *χ*_FC_ curves merge down to 50 K below which they deviate with magnitude of *χ*_FC_ greater than *χ*_ZFC_. However, the susceptibility data measured at 1 tesla field does not show any deviation between *χ*_ZFC_ and *χ*_FC_. Irrespective of the applied field strength, both *χ*_ZFC_ and *χ*_FC_ continue to increase with decrease in the temperature without any anomalies. In the absence of signatures for a typical antiferromagnet whose magnetization decreases as temperature decreases, the magnetic response may be ascribed to random arrangement of magnetic spins. These experimental findings point to the paramagnetic nature of this compound in the entire temperature range. For EuMnO_3_, the *χ*_ZFC_ and *χ*_FC_ curves merge with each other down to ∼50 K, below which *χ*_ZFC_ displayed a peak while *χ*_FC_ increased. A large bifurcation in the curves was observed at low temperatures and low fields. d*χ*_FC_/d*T* curve revealed two types of transitions as marked by arrows in the Fig. 6 around 50 K and 35 K. By comparing with literature, a small broad anomaly at <50 K may be ascribed to the onset of incommensurate antiferromagnetic ordering of Mn ions (*T*_N1_) and the low temperature anomaly around 35 K may be associated with the collinear arrangement of Mn^3+^ ions in the antiferromagnetic state (*T*_N2_). Nevertheless, magnetization at low temperatures tends to increase/saturate with lowering temperature resembling that of a ferromagnet. This may imply the existence of ferromagnetic correlations inside the antiferromagnetic state. Hence the low temperature magnetic ground state may be A-type AFM. This is corroborated with the observation of large coercive fields from *M*–*H* curves whose results will be discussed in the ensuing sections. *χ*(*T*) data of all the compounds follow Curie–Weiss behavior in the paramagnetic state. A representative CW fit to field cooled *χ*(*T*) data of EMO collected at 10 kOe is shown in Fig. 5(e). The CW fit parameters of the compounds obtained by fitting the *χ*_FC_ data measured at various fields were used to determine the values of effective magnetic moment, *μ*_eff._. Variation of *μ*_eff._ with external magnetic field is displayed in Fig. 7.

The effective magnetic moment is calculated from the atomic moments of the magnetic ions as per the chemical formula of the compounds from the relation:4
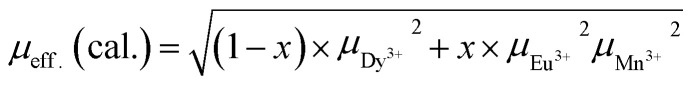


Atomic moments of Dy^3+^ (10.62 *μ*_B_), Eu^3+^ (3.46 *μ*_B_) and Mn^3+^ (4.89 *μ*_B_) are used to determine *μ*_eff._ (cal.). The values of *μ*_eff._ (cal.) thus calculated are found to reduce with increase in the europium concentration, *x* in DEMO. It may be remarked here that *μ*_eff._ (expt.) obtained from 1 tesla data is close to the calculated value (Table 3).

**Table 3:** The effective magnetic moment values of Dy_1−*x*_Eu_*x*_MnO_3_ estimated from the CW fit along with the calculated values from the eqn (4)

*x*	*μ*_B_	
	*μ*_eff._ (cal.)	*μ*_eff._ (expt.)
0	11.69	11.66
0.1	11.26	11.49
0.3	10.32	10.35
0.5	9.29	9.22
0.8	7.49	7.91
1.0	6	6.42

As discussed in the earlier sections, the origin for a series of magnetic transitions observed in DMO was attributed to antiferromagnetic coupling between Mn and Dy ions. Comparison of the experimental findings of the substituted compounds with those of the end compounds, DMO and EMO, the following conclusions can be made:

(a) The *x* = 0.1, 0.3 and 0.5 compounds with a single anomaly in the *χ*(*T*) data exhibit a magnetic transition from paramagnetic to antiferromagnetic structure.

(b) *x* = 0.8 compound does not undergo any type of magnetic ordering.

(c). EMO indicates the onset of IC-AFM for *T* ≤ 50 K and C-AFM around 35 K which agrees with the neutron diffraction measurements carried out by Ferreira *et al.*^32^ revealing A-AFM.

**Fig. 8:**
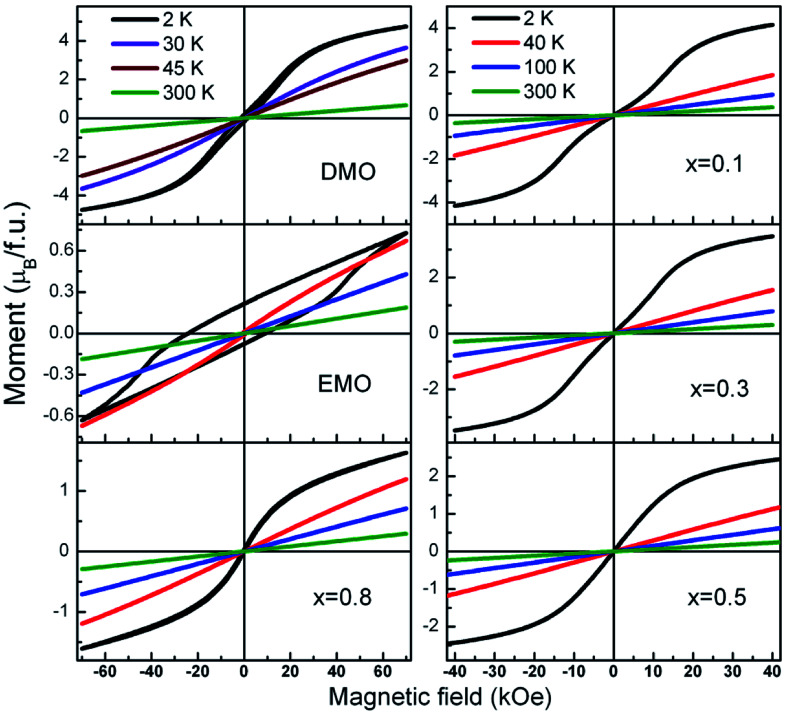
Magnetization loops a function of applied magnetic field measured at different fixed temperatures for all the europium concentrations.

**Fig. 9:**
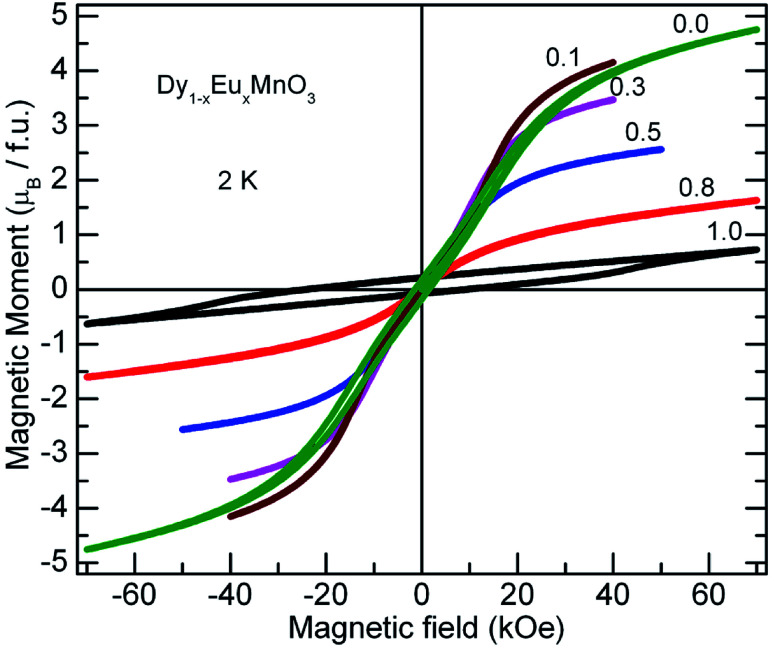
Magnetic moment (*M*) against applied magnetic field recorded at 2 K for Dy_1−*x*_Eu_*x*_MnO_3_ (*x* = 0.0–1.0). Reduction of magnetic moment by Dy sub lattice dilution with a non magnetic Eu ion is evident from the curves.

Further, to clarify the observations of the temperature dependence of magnetization data and to identify the magnetic nature of these materials, isothermal magnetization measurements at different fixed temperatures were performed. *M*–*H* curves for all the compounds at selected temperatures are presented in Fig. 8. A linear relationship between the magnetic moment and magnetic field was observed at high temperatures (300 K and 100 K) for all the compounds. It is seen from the Fig. 8, that the isothermal curves measured at 2 K for the compounds with *x* = 0.1 to 0.8 show S shape wherein the moment increases gradually in the low magnetic field region without hysteresis followed by a slope change which again increases with increase in the field strength. As temperature of the measurement increases, *M*–*H* curves show linear behavior. These S type curves have a very low remnant magnetization and do not exhibit coercivity. On the other hand, EMO has a large coercive field. Hysteresis curves of DMO and EMO with appreciable coercive fields show no sign of saturation up to 7 T. However, the remnant magnetization is small, 0.174 *μ*_B_ f.u.^−1^ and 0.214 *μ*_B_ f.u.^−1^ for DMO and EMO respectively. These features indicate the short range ferromagnetic correlations within the antiferromagnetic state (Fig. 9).

## Conclusions

Polycrystalline Dy_1−*x*_Eu_*x*_MnO_3_ (*x* = 0.0–1.0) compounds were prepared by solid state reaction. All the compounds crystallized in the orthorhombic structure. Contributions from the atomic vibrations to the observation of Raman bands have been established and assigned to symmetry stretching and anti symmetry stretching, bending and tilting modes. Raman band frequencies of the tilting and bending modes in DEMO decreased with increase in europium content showing softening. The transport results revealed that all the compounds show semiconducting nature. All the substituted compounds showed activated type of conduction. EMO showed VRH type conduction. Hopping energy (*E*_hop._) is calculated and it is observed that the activated energy was found to reduce. In contrast to the parent compound, DMO, all DEMO compounds showed only one type of magnetic transition *i.e.*, antiferromagnetic nature corresponding to Mn ions. The magnetic field dependent magnetization at various temperatures revealed paramagnetic nature down to 8 K below which hysteresis loops are observed. The presence of strong ferromagnetic correlations between Dy and Mn spins through apical oxygen ions results in the large coercive fields. For temperatures above the antiferromagnetic temperature of manganese ions (39 K) *M*–*H* curves show almost straight lines implying an absence of any ferromagnetic interactions in the compounds. Different magnetic transitions: from high temperature paramagnetic state to intermediate temperature antiferromagnetic state to low temperature ferromagnetic states are observed in the *M*–*H* data.

## Conflicts of interest

There are no conflicts to declare.

## Supplementary Material
